# Glucocerebrosidase inhibition causes mitochondrial dysfunction and free radical damage

**DOI:** 10.1016/j.neuint.2012.10.010

**Published:** 2013-01

**Authors:** Michael W.J. Cleeter, Kai-Yin Chau, Caroline Gluck, Atul Mehta, Derralynn A. Hughes, Michael Duchen, Nicholas William Wood, John Hardy, J. Mark Cooper, Anthony Henry Schapira

**Affiliations:** a Department of Clinical Neurosciences, University College London Institute of Neurology, United Kingdom; b Lysosomal Storage Disorders Unit, University College London, Department of Hematology, United Kingdom; c University College London, Department of Cell and Developmental Biology, United Kingdom; d Department of Molecular Neuroscience, University College London Institute of Neurology, United Kingdom

**Keywords:** Parkinson’s disease, Glucocerebrosidase, Autophagy, Mitochondria, Oxidative stress, Free radicals, Alpha-synuclein, Membrane potential, Neurodegeneration, Gaucher disease

## Abstract

Mutations of the gene for glucocerebrosidase 1 (*GBA*) cause Gaucher disease (GD), an autosomal recessive lysosomal storage disorder. Individuals with homozygous or heterozygous (carrier) mutations of *GBA* have a significantly increased risk for the development of Parkinson’s disease (PD), with clinical and pathological features that mirror the sporadic disease. The mechanisms whereby *GBA* mutations induce dopaminergic cell death and Lewy body formation are unknown. There is evidence of mitochondrial dysfunction and oxidative stress in PD and so we have investigated the impact of glucocerebrosidase (GCase) inhibition on these parameters to determine if there may be a relationship of *GBA* loss-of-function mutations to the known pathogenetic pathways in PD. We have used exposure to a specific inhibitor (conduritol-β-epoxide, CβE) of GCase activity in a human dopaminergic cell line to identify the biochemical abnormalities that follow GCase inhibition. We show that GCase inhibition leads to decreased ADP phosphorylation, reduced mitochondrial membrane potential and increased free radical formation and damage, together with accumulation of alpha-synuclein. Taken together, inhibition of GCase by CβE induces abnormalities in mitochondrial function and oxidative stress in our cell culture model. We suggest that *GBA* mutations and reduced GCase activity may increase the risk for PD by inducing these same abnormalities in PD brain.

## Highlights

► Glucocerebrosidase gene mutations are a risk factor for Parkinson’s disease. ► Glucocerebrosidase inhibition causes mitochondrial dysfunction & oxidative stress. ► These changes parallel important pathogenetic of Parkinson’s disease.

## 1 Introduction

Glucocerebrosidase 1 (GCase) is a ubiquitous lysosomal enzyme responsible for the breakdown of glucocerebroside to glucose and ceramide. Diverse mutations within the gene (*GBA*) that encodes GCase result in mutant enzymes with reduced activity and an autosomal recessive storage disorder (Gaucher disease – GD). GD patients have reduced GCase activity while heterozygote carriers generally have an intermediate level (Raghavan et al., 1980). GD is characterised by widespread accumulation of the GCase substrates glucocerebroside or glucosylsphingosine in many organs (Grabowski, 2008). Although *GBA* mutations cause a reduction in enzyme activity, this may not necessarily be the mechanism that mediates the pathogenesis of GD and alternative models include mis-trafficking of GCase and endoplasmic reticulum stress (Kov-Bar et al., 2011).

Alpha-synuclein positive Lewy bodies have been identified in the brains of GD patients and carriers who died with PD (Neumann et al., 2009; Wong et al., 2004). There are now persuasive data that *GBA* mutations are a major risk factor for PD and result in a clinical and pathological phenotype that is virtually indistinguishable from sporadic PD (Sidransky et al., 2009).

The mechanism(s) whereby *GBA* mutations increase the risk for PD remain unidentified. PD pathogenesis is thought to involve a number of pathways including mitochondrial dysfunction and oxidative stress (Schapira, 2006). Given the similar clinical and pathological phenotypes of *GBA*–PD and sporadic PD, we hypothesised that reduced GCase activity would result in biochemical events that would map to these same pathways considered of pathological relevance to familial and sporadic PD. We therefore investigated the effects of a specific GCase inhibitor (conduritol-β-epoxide) on mitochondrial function and free radical generation.

## 2 Materials and methods

### 2.1 Reagents

Reagents were supplied by Sigma–Aldrich (Poole, UK) and Merck (Nottingham, UK) unless otherwise stated.

### 2.2 Cell cultures and treatments

SHSY-5Y cells were maintained as described (Alvarez-Erviti et al., 2010). During the course of continuous conduritol-β-epoxide (CβE; Universal Biologicals, Cambridge, UK) treatment, cells were split 1:3 every 3 days with fresh CβE added to a final concentration of 50 μM. GCase activity was monitored at different times throughout the course of the experiment to check that inhibition was maintained. Cell viability was checked by lactate dehydrogenase (LDH) release assays (Roche, UK). In brief, cells were plated into pairs of wells of a 48-well dish in phenol red-free media. After 48 h incubation of the control and CβE treatment, Triton X-100 was added to the medium of one of each pair of wells to a final concentration of 1% and the LDH level in the medium served as its total level. The LDH level found in the medium of the other well represents released LDH. Results were expressed as percentage LDH release from the total. For other assays, cells were harvested by trypsinisation. If required for assay of GCase, mitochondrial respiratory chain (MRC), or aconitase activities, cells were centrifuged at 1000 rpm, washed twice with phosphate-buffered saline (PBS) and stored as a pellet at −80 °C until assayed. Cells were then resuspended in 0.25 mM sucrose, 50 mM Tris (hydroxymethyl) aminomethane hydrochloride (Tris–HCl) pH 7.4 and freeze–thawed three times prior to assay. If required for adenosine diphosphate (ADP) phosphorylation measurements, cell numbers were counted using haemocytometers (Immune Systems; Paignton, UK), and cells were centrifuged and resuspended in buffer for assays (see below).

### 2.3 Enzyme assays and ATP synthesis measurements

Respiratory chain (Schapira et al., 1990), proteasome (Schapira et al., 2006), aconitase (Bradley et al., 2000) and ADP phosphorylation activities (Korlipara et al., 2004) were measured by standard techniques as described previously. CβE-sensitive GCase activity (end-point measurement) was determined at 37 °C essentially as described (Prence et al., 1996) using 4-Methylumbelliferyl-β-d-glucopyranoside as substrate in a plate reader (‘Synergy’, LabTech; Brighton, UK). The increase in fluorescence of released 4-Methylumbelliferone at 460 nm following excitation at 360 nm was followed over 1 h. Protein levels were estimated using a bicinchoninic acid (BCA) kit (Pierce Thermo Fisher; Basingstoke, UK) with reference to the protein standard supplied with the kit.

### 2.4 Western blot analysis

Cells were harvested, washed with PBS and processed as described (Alvarez-Erviti et al., 2010). 25–40 μg of whole cell lysates were electrophoresed on Novex gels (NuPage 4–12%; Invitrogen, Paisley, UK) and transferred onto polyvinylidene fluoride membrane (Millipore; Watford, UK) and then probed with antibodies to porin (Merck; Darmstatd, Germany; 1/25000 dilution), adenine nucleotide transporter (ANT; Abcam, UK, 1/1000 dilution), microtubule-associated protein 1 light chain 3 (LC3, Clone D11; Cell Signalling, USA, 1/1000 dilution), alpha-synuclein (Becton–Dickinson, UK, 1/500 dilution), Glucocerebrosidase (GBA; Abcam, UK, 1.1000 dilution) or lysosome-associated membrane proteins (lamp1, clone H4A3; Abcam, UK, 1/1000 dilution) and all were normalised to β-actin (Abcam; 1/5000 dilution) Blots were developed using an enhanced chemiluminescence (ECL) kit (GE Healthcare; Little Chalfont, UK), exposed to X-ray film (GE Healthcare). The film was developed and signal intensities in the linear range were quantified by the ‘Alphadigidoc’ software package (AlphaInnotech; San Leandro, USA).

### 2.5 Live cell confocal imaging and analysis

Fluorescence of cells grown on 22 mm coverslips were measured by real-time confocal imaging as described (Gandhi et al., 2009; Duchen et al., 2003), using a Zeiss 510 laser scanning microscope equipped with an additional Enterprise UV laser source and a cooled charge coupled device camera, bathed in standard phenol red-free Hank’s Buffered Salt Solution at room temperature. Mitochondrial membrane potential (*Ψ*_m_) was quantified by steady-state fluorescence (excited using the 543 nm laser line and measured using a 560 nm longpass filter) of mitochondrial patterns produced by 25 nM tetramethyl rhodamine methyl ester (TMRM; Invitrogen) stained at room temperature for 45 min. Free radical generation was measured by the rate of ratiometric change of reduced and oxidised dihydroethidium (DHE; Invitrogen) fluorescence. 10 μM of DHE was loaded at room temperature, and measurements were made typically over 120 s. Oxidised DHE was measured with the 543 nm laser line and 560 nm long-pass filter, while for reduced DHE measurement it was excited at 351 and measured at 435–485 nm. Cells were treated accordingly and measured at the same time to minimise variability of fluorescence measurements. Individual cells were marked and mean fluorescence of individual cells measured by the ImageJ software (NIH, USA); where the image was captured with *Z*-stack, *z*-projection was performed using max intensity and net fluorescence was obtained by subtracting the background fluorescence. Mitochondrial morphology was measured on the circularity and aspect ratio from the TMRM images by ImageJ as described (Wang et al., 2011).

### 2.6 Creation of *GBA* knockdown SHSY-5Y stable cell lines

SHSY-5Y cells were transfected with a ‘Hush’ GBA knockdown plasmid (Origene, USA), empty plasmid and scrambled control (The sequence chosen for the *GBA* knockdown was: GTGTGTGTCTGCAATGCCACATACTGTGA). Stable clones were isolated following selection with puromycin (Sigma, UK) at 4 μg/ml and characterised by analysis of GCase activity, actin-normalised *GBA* mRNA by a ‘StepOne’ QPCR machine (Applied Biosystems, UK) using SyBr Green (Life Technologies, UK) and appropriate primers for *GBA* and β-actin (Eurofins, Germany) and GCase protein levels (by Western blotting). Clones were assessed after several passages (in the presence of a maintenance dose of 2 μg/ml puromycin) to check for the continuation of any knockdown effect.

### 2.7 Statistical analysis

Where multiple comparisons were made, one-way ANOVA tests were performed followed by Dunnett post test analysis in order to determine statistical significance. Student’s *t*-tests were used for comparing statistical significance between 2 populations. A *p* value of < 0.05 was considered as significantly different.

## 3 Results

### 3.1 CβE

CβE has been reported to be a selective inhibitor of GCase activity (Prence et al., 1996; Newburg et al., 1986) and we have confirmed in SHSY-5Y cells that 50 μM CβE decreased GCase activity to ⩽5% of untreated cells and maintained the inhibition of GCase activity over 30 days (Suppl. Fig. 1). This concentration of CβE has also been previously reported to result in a greater than 2-fold increase of glucocerebroside over 24 days (Prence et al., 1996). In our experiments, 30 days CβE treatment had no effect on cell viability as judged by LDH release (Suppl. Fig. 2).

### 3.2 Mitochondrial studies

#### 3.2.1 ATP synthesis (ADP phosphorylation)

**Fig. 1:**
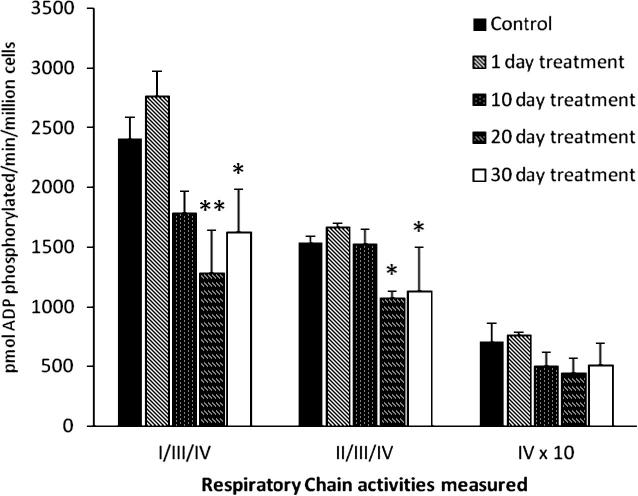
CβE treatment led to a reduction in ADP phosphorylation. Glutamate (complexes I, III and IV) and succinate-dependent (complexes II, III and IV) ADP phosphorylation was significantly impaired following incubation with CβE for 20 days onwards. Ascorbate/TMPD-dependent (complex IV) ADP phosphorylation was not significantly altered. Solid bars: control; diagonal line bars: 1 days incubation; Dotted bars: 10 days incubation; horizontal striped bars: 20 days incubation; open bars: 30 days incubation. Activities (mean ± SEM) were corrected by cell number, measured from 3 experimental repeats. One-way ANOVA analysis (followed by Dunnett post test) was performed on the data and showed that both glutamate and succinate-linked activities were decreased significantly from 20 days onwards (^∗^*p* < 0.05, ^∗∗^*p* < 0.01).

Fig. 1 shows the ADP phosphorylation capacity of digitonin-permeabilised cells following incubation with CβE. There was no measurable effect before 10 days, but complex I-linked ADP phosphorylation with glutamate/malate as substrate was significantly decreased by 47% at 20 days (*p* < 0.01) and by 33% at 30 days (*p* < 0.05), compared to control. Complex II/III-linked ADP phosphorylation using succinate as substrate was significantly reduced by 30% at 20 days (*p* < 0.05) and 26% at 30 days (*p* < 0.05). There was no significant change in complex IV-linked ADP phosphorylation with ascorbate/TMPD as substrate. Basal levels of ATP (determined from time zero acid-treated samples) were 616 ± 149 and 643 ± 270 pmol/million cells, for control and 30 day treated cells respectively. These values represented approximately 1.5–4.5% of the values determined for ADP phosphorylation with glutamate/malate over the incubation period.

#### 3.2.2 Respiratory chain activities

There was no statistically significant effect of CβE on the function of individual isolated respiratory chain complex enzymatic activities (Table 1)

#### 3.2.3 Mitochondrial membrane potential (*Ψ*_m_)

**Fig. 2:**
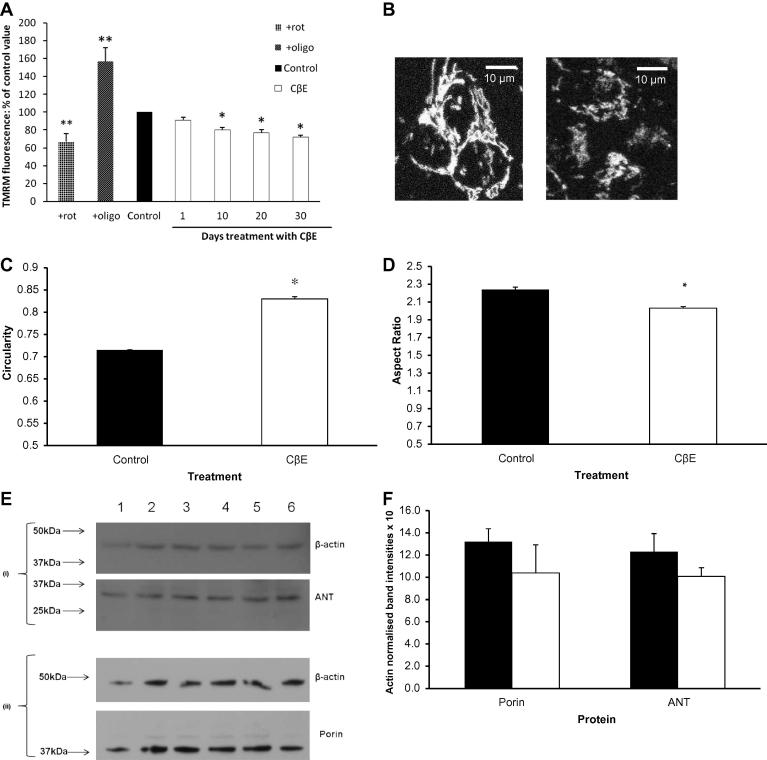
CβE treatment led to a progressive fall in mitochondria membrane potential (*Ψ*_m_) and no change in mitochondrial content. (A) Deflections in the system are demonstrated by the use of rotenone (cross-hatched bar; ‘+rot’) and oligomycin (diagonal shaded bar; ‘+oligo’); ^∗∗^*p* < 0.001. Data (mean ± SEM) for each time point where 195 cells from 5 coverslips representing 3 independent preparations were analysed, and is expressed as percentage changes (open bars) to the untreated control (solid bar). 50 μM CβE produced a significant and progressive fall in *Ψ*_m_ from 10 days (^∗^*p* < 0.01 after ANOVA followed by Dunnett post test analysis). (B) Representative TMRM fluorescence images of untreated and SHSY-5Y cells treated with CβE for 30 days showing disruption of the mitochondrial network. (C) Image analysis reveals that both the circularity and (D) aspect ratio of the mitochondria upon CβE treatment are significantly increased (^∗^*p* < 0.0001 by *t*-test), suggesting the mitochondria are more fragmented (solid bars: control; open bars: treated cells. (E) Levels of porin and ANT were measured in SHSY-5Y cells incubated with 50 μM CβE for 30 days (lanes 4–6) by Western blot analysis, against untreated cells (lanes 1–3) Blot (i) upper panel: β-actin; lower panel: ANT. Blot (ii) upper panel: β-actin; lower panel: porin. Graph (F) shows that there was no significant effect on porin or ANT levels (mean ± SEM, *n* = 3) by this treatment, (black bars, control; open bars: treated cells).

Fluorescence measurements of TMRM staining at nM level at its steady-state produces mitochondrial staining patterns and they are a reliable indicator of mitochondrial membrane potential in live cells (Duchen et al., 2003). The sensitivity of the measurement was validated by the complex I inhibitor rotenone, which reduced *Ψ*_m_ and TMRM fluorescence, while the ATPase inhibitor oligomycin increased *Ψ*_m_ and TMRM fluorescence (Fig. 2A).

There was no acute effect of CβE on TMRM staining and *Ψ*_m_ after 1 day, indicating there was no direct effect of the compound on mitochondrial function or any quenching effect on fluorescence (Fig. 2A). CβE induced a progressive and significant fall in *Ψ*_m_ by 20% at 10 days (*p* < 0.01), 23% at 20 days (*p* < 0.01) and 28% at 30 days (*p* < 0.01). We also observed fragmentation of the mitochondrial network at 30 days (Fig. 2B–D).

#### 3.2.4 Mitochondrial content

Western blot analysis of the mitochondrial marker proteins porin and adenine nucleotide transporter (ANT), normalised to actin as a loading control, showed no significant change with CβE. This indicates that CβE treatment for up to 30 days had no significant effect on mitochondrial content (Fig. 2E and F).

### 3.3 Oxidative stress

**Fig. 3:**
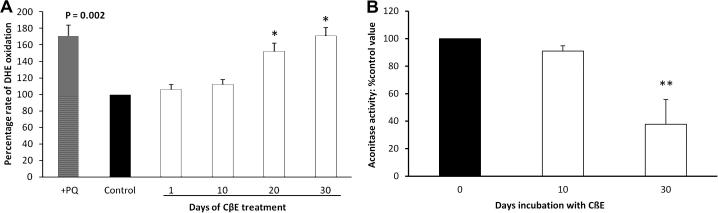
CβE treatment led to increased free radical production and reduced aconitase activity. (A) As a positive control, paraquat pre-treatment at 300 μM for 1 day (cross-hatched bar; ‘+PQ’) significantly increased the rate of DHE oxidation, as analysed by paired *t*-tests (*p* = 0.002). Continuous treatment of SHSY-5Y cells with 50 μM CβE (open bars) showed a progressive increase in the rate of DHE oxidation after 20 days (^∗^*p* < 0.01, mean percentage changes with respect to the untreated control (solid bar) ±SEM) as determined by one-way ANOVA followed by Dunnett post test. (B) Aconitase activity was significantly reduced (to 37% of control) after 30 days of CβE treatment (mean ± SEM, ^∗∗^*p* < 0.001).

DHE fluorescence was used to measure reactive oxygen species generation in live cells, for example as demonstrated for the free radical generator paraquat (Fig. 3A). CβE caused a significant increase in the rate of DHE oxidation by 52% at 20 days (*p* < 0.01) and by 71% at 30 days (*p* < 0.01). Aconitase activity, a measure of free radical mediated damage, was reduced by 60% (*p* < 0.001) after 30 days incubation with CβE (Fig. 3B).

### 3.4 Protein degradation studies

#### 3.4.1 (A) Ubiquitin Proteasomal System (UPS)

**Fig. 4:**
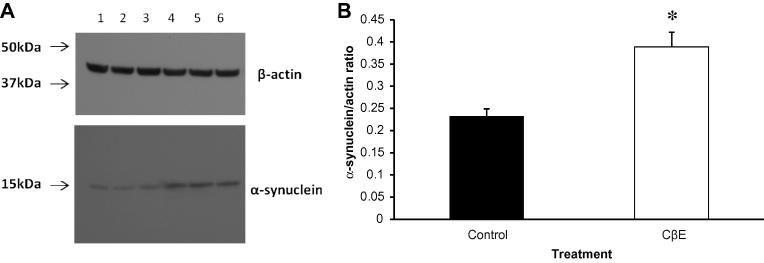
CβE treatment led to increased alpha-synuclein. Levels of alpha-synuclein were measured in SHSY-5Y cells incubated with 50 μM CβE for 30 days by Western blot analysis, against untreated cells. (A) Shows the blots of untreated (lanes 1–3) or CβE-treated (lanes 4–6) SHSY-5Y cells, stained with anti-alpha-synuclein (lower panels) or anti-β-actin (upper panels). (B) Shows the graphical representation of alpha-synuclein levels from the scanned blots, normalised to β-actin levels (solid bar: control, open bar: treated cells). The level of alpha-synuclein protein was significantly (^∗^*p* < 0.002; *n* = 3) higher (by 59%) than that of control levels.

The accumulation of alpha synuclein and other proteins in Lewy bodies is a characteristic feature of PD pathology. The UPS has been reported to be abnormal in PD brain and therefore potentially to contribute to protein aggregation (McNaught and Jenner, 2001). We investigated the effect of GCase inhibition on proteasomal function, but found no abnormalities (Table 1). We did observe a significant increase (59%, *p* < 0.002) in alpha synuclein levels in the treated cells, as determined by Western blotting (Fig. 4A and B). Using alpha-synuclein fused to green fluorescent protein (GFP), at 20 days there was a 49% increase in levels as reflected by fluorescence in SHSY-5Y cells following CβE (Suppl. Fig. 3).

#### 3.4.2 (B) Lysosomal studies

**Fig. 5:**
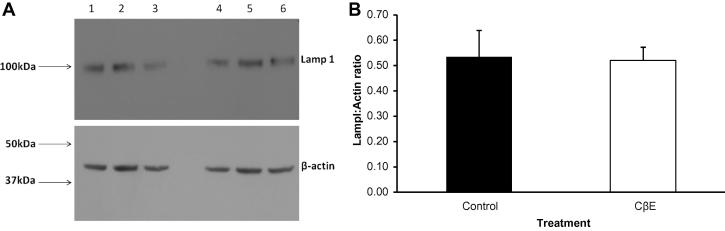
CβE treatment did not affect Lamp 1 levels. Levels of Lamp 1 were estimated by Western blotting of control cells (lanes 1–3) and treated ones (lanes 4–6) with anti-Lamp 1 and normalising with anti-β-actin. (A) Shows the blot and (B) shows a graphical representation of the result (solid bar: control, open bar: treated). No significant difference was seen between the two groups (*n* = 3).

**Fig. 6:**
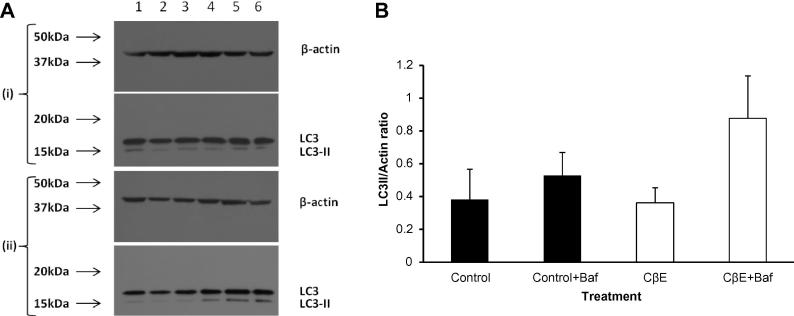
CβE treatment did not significantly alter basal LC3-II levels. LC3-II levels were estimated for treated and control cells. CβE did not affect the basal levels of LC3-II. (A): Shows Western blots of control and CβE cells (blot (i): without bafilomycin; blot (ii): with bafilomycin); control (lanes 1–3) or CβE (lanes 4–6). (B): Shows a graphical representation of the result (solid bars: control, open bars: CβE treated). The levels did not change significantly after CβE treatment.

An indication that the lysosomal content of the cells was unchanged is shown in Fig. 5A and B: Lamp1 levels (normalised to actin) showed no significant difference between control cells and CβE treated ones. Western blots of LC3-II on SHSY-5Y cells did not show a change in the levels of LC3-II when GCase was inhibited by CβE (Fig. 6A and B). Bafilomycin treatment (100 nM for 3 h) of these cells did not appear to increase the observed levels significantly).

### 3.5 *GBA* knockdown

To confirm the effects of GCase inhibition by CβE, we generated a stable shRNA-mediated knockdown model of *GBA* in SH-SY5Y cells. Suppl. Fig. 4A shows that the enzyme activity was reduced by 62% and Western blot band densities indicated that the level of protein was decreased by 59% (Suppl. Fig. 4B and C), compared to the scrambled control levels. Quantitative PCR data also showed a significant decrease of 60% in the mRNA for *GBA* relative to the scrambled control (data not shown). As shown in Suppl. Fig. 4D, knockdown of *GBA* caused a significant fall in TMRM fluorescence (*p* < 0.05) compared to the controls i.e. parental SHSY-5Y and cells stably expressing the scrambled shRNA. There was a 38% reduction (*p* = 0.003) in aconitase activity (Suppl. Fig. 4E). An increase in alpha synuclein was seen in the knockdown cells, although this just failed to reach significance (*p* < 0.067); Suppl. Fig. 5A and B). As seen with the chemical model, the GCase knockdown cells did not show a significant increase in the basal LC3-II levels or when the cells were treated with bafilomycin (100 nM, 3 h) (Suppl. Fig. 6A and B).

### 3.6 Other models of lysosomal inhibition

Inhibition of lysosomal function by bafilomycin or ammonium chloride produced a different pattern of impaired ADP phosphorylation (bafilomycin) or no effect (ammonium chloride) (Suppl. Fig. 7A). These inhibitors also showed different effects to CβE on mitochondrial membrane potential (Suppl. Fig. 7B). These results support our data that the mitochondrial effects of CβE are not mediated directly through lysosomal inhibition.

## 4 Discussion

The aetiopathogenesis of PD is thought to involve an interaction of genetic and environmental factors and includes mitochondrial dysfunction, oxidative stress and protein handling abnormalities, as well as inflammation (Schapira and Tolosa, 2010; Schapira, 2012). Specific gene mutations have been identified as causes of familial PD and genome-wide association studies have highlighted the importance of alpha-synuclein and tau as contributors to PD risk. The genetic causes of familial PD are thought to initiate biochemical events that map to these same pathways. *GBA* mutations have now been reproducibly associated with a substantially increased risk for PD estimated variously as 5 to 20-fold (Sidransky et al., 2009; Bultron et al., 2010).

We have followed over time the effects of GCase enzyme inhibition and knockdown on mitochondrial function and oxidative stress. In our cell model, the first change in function we observed following CβE exposure was a progressive decline in mitochondrial membrane potential that reached significance at 10 days. Our studies then showed that inhibition of GCase activity to a degree comparable to that seen in GD (Raghavan et al., 1980), caused a significant reduction of ATP synthesis involving both complex I-linked and complex II/III- linked ADP phosphorylation although the individual respiratory chain protein activities were unaffected. This deficiency was seen after 20 days incubation, suggesting that the reduction in ATP synthesis was neither a direct result of CβE on some cellular component, nor due to the immediate effects of GCase inhibition but rather mediated via pathways resulting from GCase inhibition. By 20 days there was also a significant and progressive increase in free radical generation and this was maintained to 30 days, at which point there was also a significant reduction in aconitase activity, an indicator of free-radical mediated damage. The fragmentation of mitochondria seen in cells exposed to CβE corresponds with the fall in mitochondrial membrane potential that is also observed on exposure to this toxin. It will be of interest to investigate the function of mitophagy and mitochondrial biosynthesis in this model, and this is underway. It is possible that the increased oxidative stress identified in this model may be explained in part by an accumulation of defective mitochondria. The UPS activities were not affected in our CβE model, but there was evidence of accumulation of alpha-synuclein.

These results indicate that inhibition of a lysosomal enzyme, GCase, causes abnormalities of mitochondrial function and oxidative stress, pathways that are also known to be defective in PD brain. A defect of ATP synthesis but with normal individual respiratory chain enzyme activities can be seen in certain mitochondrial diseases and suggests a defect of electron transfer or mitochondrial membrane defect (Schapira, 2012). Although CβE is considered a specific inhibitor of GCase, we cannot completely exclude the possibility of off-target effects or secondary pathways initiated by GCase inhibition inducing the effects seen. However, *GBA* knockdown leading to a 62% reduction in enzyme activity induced a similar pattern of changes with a decrease in mitochondrial membrane potential and a significant fall in aconitase activity. Furthermore, the increase in oxidative stress observed here is compatible with a previous observation from GD fibroblast lines of an increase in superoxide ions generated from non-phagocytic NADPH oxidase and an associated increase in protein oxidation in the form of protein carbonyls (Deganuto et al., 2007).

Recent data have supported a role for defective lysosomal-dependent degradation (autophagy) in PD. Abnormalities of the chaperone-mediated autophagy (CMA) pathway have been demonstrated in PD brain in anatomic regions that map to neuronal degeneration and Lewy body deposition (Alvarez-Erviti et al., 2010). *GBA* mutations would be expected to reduce GCase activity in the lysosome and lead to the accumulation of glucocerebroside and glucosylsphingosine. A direct interaction between GCase and alpha-synuclein under lysosomal conditions has recently been described (Yap et al., 2011). It is notable that *GBA*–PD pathology is alpha-synuclein Lewy body-positive, and alpha-synuclein is predominantly metabolised by CMA. The accumulation of alpha synuclein in *GBA*-associated PD may therefore reflect reduced turnover secondary to impaired CMA or lysosomal function. Knockdown of GCase has been shown to reduce the rate of proteolysis in cells by 40% through disruption of the lysosomal pathway (Mazzulli et al., 2011). Alpha synuclein steady state levels were increased in GD cells and GCase knockout models with an accumulation of a soluble high molecular weight form of the protein. Alpha synuclein aggregates have also been seen in a mouse model of GD (Sardi et al., 2011). It is of interest therefore that CβE inhibition of GCase activity in our cell model caused an increase in alpha-synuclein levels. This has also been reported following CβE treatment in mouse substantia nigra (Manning-Bog et al., 2009). We have recently studied post-mortem brain samples from PD patients with and without *GBA* mutations (Gegg et al. in press). In addition to reduced GCase activity in both *GBA* positive and negative brains, most profound in substantia nigra, we demonstrated *in vivo* and *in vitro* features supportive of the reciprocal relationship between alpha-synuclein and GCase activity. Thus, the lower the activity of GCase, the higher the alpha-synuclein levels, and the greater the expression of alpha-synuclein the lower the GCase activity.

The results from the present study indicate that inhibition of GCase induces defects in mitochondrial function, increases oxidative stress and confirms previous reports that inhibition of this enzyme increases alpha-synuclein levels. These abnormalities are also found in the PD brain, particularly in the substantia nigra, the main site of neuronal degeneration. We suggest that *GBA* mutations and decreased GCase activity increase the risk for PD by inducing or exacerbating these same abnormalities of mitochondrial function, oxidative stress and alpha-synuclein accumulation to cause or accelerate the development of PD.

## Figures and Tables

**Table 1:** Mitochondrial respiratory chain and proteasome activities following CβE treatment of SHSY-5Y cells for 30 days.

Assay	Control	30 days CβE
(*a*) *MRC activities*		
CXI/CS × 100	4.3 ± 1.1	3.3 ± 1
CXII/III/CS × 1000	13.4 ± 0.6	14.6 ± 3.1
CXIV/CS × 1000	4.3 ± 1.3	4.6 ± 0.8

(*b*) *Proteasome activities*⁎		
PGP-like	1.6 ± 0.6	2.5 ± 0.25
Chymotrysin-like	6.0 ± 1.8	5.7 ± 2.0
Trypsin-like	8.7 ± 2.9	8.6 ± 1.4

⁎ mFluorescence units/min/mg protein × 10^−6^.

## Appendix A Supplementary data

Supplementary data 1 Incubation of SHSY-5Y cells with CβE caused a continuous inhibition of GCase. Fresh medium plus 50 μM of inhibitor added, as indicated by arrows, upon splitting of the cells every 3–4 days over a 30 day period caused a >95% inhibition of GCase. This inhibition remained constant throughout the regime.

Supplementary data 10 Influence of other lysosomal inhibitors on mitochondria. SHSY-5Y cells stably expressing green fluorescent protein (GFP) in the mitochondria were treated with 200 nM of bafilomycin for 24 h, or 20 mM of ammonium chloride (NH4Cl) for 7 days respectively. Graph (A) shows the results of ADP phosphorylation assays (solid bars: control, diagonal shaded bars: bafilomycin treated cells, and spotted bars: NH4Cl treated cells; activities corrected by protein level). Graph (B) shows the measurement of GFP fluorescence that represents mitochondrial content, and mitochondrial membrane potential (*Ψ*_m_) that was calculated from TMRM fluorescence corrected by mitochondrial content (solid bars: control, diagonal shaded bars: bafilomycin, spotted bars: NH4Cl). Shown in the graphs are means ± SEMs as percentages of the control values. Six independent ADP phosphorylation assays were performed, and fluorescence was measured on 20 cells. ^∗^*p* = 0.001, ^∗∗^*p* = 0.0001, ^∗∗∗^*p* < 0.0001 upon Student’s *t*-tests. GFP was measured with the 488 nm laser line and 505–550 nm long-pass filter.

Supplementary data 2 CβE treatment had no significant effect on cell viability as judged by LDH release. Following CβE treatment for 30 days, cells were assessed for cell death by the LDH assay (solid bar: control, open bar: treated). No significant difference was seen between control and CβE cells.

Supplementary data 3 CβE treatment led to accumulation of alpha-synuclein. Stable ectopic expression of a GFP-alpha-synuclein fusion (kindly provided by Dr. J. George, University of Illinois) was established in SHSY-5Y cells, and these cells were treated with 10 μM retinoic acid for 5 days, followed by 50 μM CβE for 10 and 20 days. GFP fluorescence was measured from 7 cells, and the mean ± SEM are shown. Statistical significance (^∗^*p* = 0.01) was found in GFP fluorescence between the 20 days CβE treatment (open bar on the right) and no treatment (solid bar), by Student’s *t*-tests.

Supplementary data 4 GBA knockdown led to a fall in mitochondria membrane potential (*ψ*_m_) and decreased aconitase activity. (A): GCase activity, as measured for GBA scrambled shRNA control (GBA-scr, solid bar) and a knockdown sequence (GBA–KD, open bar); a statistically significant reduction in GCase activity was seen with the knockdown (^∗^*p* < 0.02; *n* = 3).

Supplementary data 5 GBA knockdown led to a fall in mitochondria membrane potential (*ψ*_m_) and decreased aconitase activity. (B): Protein levels were estimated by Western blot, using GBA antibody and β-actin antibody as a loading control. Upper bands: GBA, lower bands: β-actin; bands 1–3 GBA-scr, bands 4–6 GBA–KD. (C): Shows a graphical representation of the blot (solid bar: scrambled control, open bar: knockdown), the GBA–KD being reduced significantly (^∗^*p* < 0.003, *n* = 3).

Supplementary data 6 GBA knockdown led to a fall in mitochondria membrane potential (*ψ*_m_) and decreased aconitase activity. (D): Control SHSY-5Y (diagonal bars), GBA-scr (solid bars) and GBA–KD cells (open bars), each 60 cells from 3 coverslips representing 2 independent preparations, were analysed to produce the mean ± SEM TMRM fluorescence. Upon ANOVA followed by Tukey-Kramer Multiple Comparisons Test, that of GBA–KD is statistically significant (^∗^*p* < 0.05) compared to the other 2 controls.

Supplementary data 7 GBA knockdown led to a fall in mitochondria membrane potential (*ψ*_m_) and decreased aconitase activity. (E): Influence of reduction in GCase activity on aconitase activities in GBA-scr (solid bars) and GBA–KD cells (open bars). The activity of aconitase was reduced compared to control by a statistically significant amount of 38% (^∗^*p* = 0.003, *n* = 3).

Supplementary data 8 GBA knockdown effect on alpha-synuclein levels. Levels of alpha-synuclein were measured in SHSY-5Y GBA knockdown and scrambled cells by Western blot analysis. (A) Shows the blots of scrambled control (GBA-scr, lanes 1–3) or knockdown (GBA–KD, lanes 4–6) cells, stained with anti-alpha synuclein (lower panels) or anti-β-actin (upper panels). (B) Shows the graphical representation of alpha-synuclein levels from the scanned blots, normalised to β-actin levels (solid bars: scrambled control, open bars: knockdown). The level of alpha-synuclein protein was non-significantly (*p* < 0.067; *n* = 3) higher (by 32%) over that of control.

Supplementary data 9 Basal LC3Il levels for GBA–KD were not significantly changed by GBA knockdown. LC3-II levels were estimated for knockdown and control cells. (A): Knocking down GCase activity did not affect the basal levels of LC3-II; (blot (i): without bafilomycin; blot (ii): with bafilomycin); control (GBA-scr, lanes 1–3) or knockdown (GBA–KD, lanes 4–6). (B) Shows a graphical representation of the blots (solid bars: scrambled control, open bars: knockdown).
