# Supplementary figures and images for: Glucocerebrosidase inhibition causes mitochondrial dysfunction and free radical damage
Source: Neurochem Int. 2013 Jan;62(1):1–7. doi: 10.1016/j.neuint.2012.10.010 (PMC3550523; doi:10.1016/j.neuint.2012.10.010)

Suppl Fig. 1

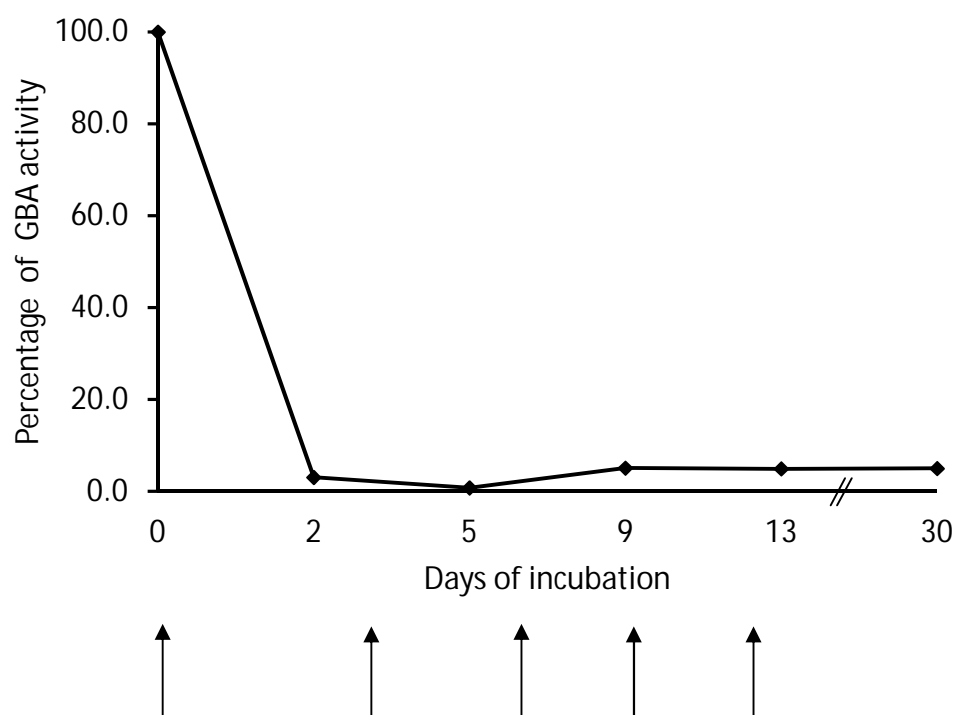

Supplement: Supplementary data 1 — Incubation of SHSY-5Y cells with CβE caused a continuous inhibition of GCase. Fresh medium plus 50 μM of inhibitor added, as indicated by arrows, upon splitting of the cells every 3–4 days over a 30 day period caused a >95% inhibition of GCase. This inhibition remained constant throughout the regime. [file mmc1.pdf]

Suppl Fig.7A

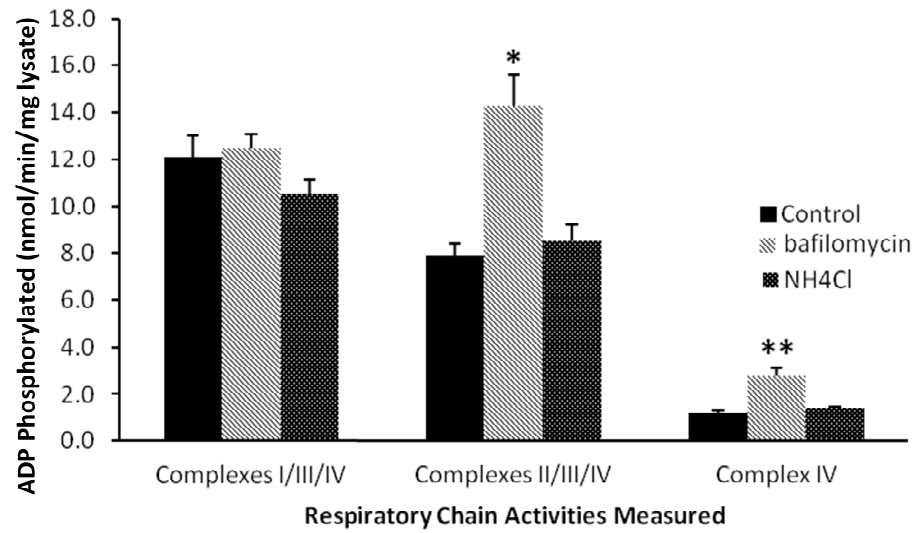

Suppl Fig.7B

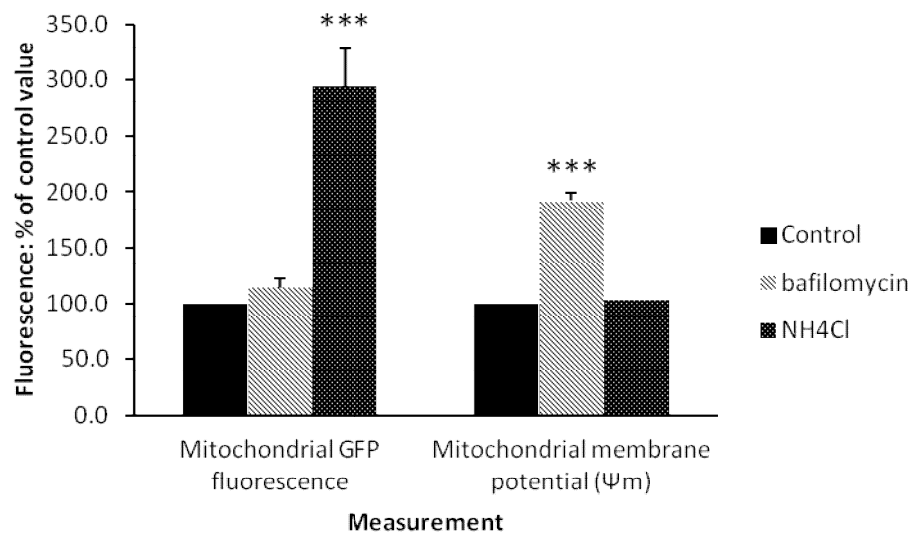

Supplement: Supplementary data 10 — Influence of other lysosomal inhibitors on mitochondria. SHSY-5Y cells stably expressing green fluorescent protein (GFP) in the mitochondria were treated with 200 nM of bafilomycin for 24 h, or 20 mM of ammonium chloride (NH4Cl) for 7 days respectively. Graph (A) shows the results of ADP phosphorylation assays (solid bars: control, diagonal shaded bars: bafilomycin treated cells, and spotted bars: NH4Cl treated cells; activities corrected by protein level). Graph (B) shows the measurement of GFP fluorescence that represents mitochondrial content, and mitochondrial membrane potential (Ψm) that was calculated from TMRM fluorescence corrected by mitochondrial content (solid bars: control, diagonal shaded bars: bafilomycin, spotted bars: NH4Cl). Shown in the graphs are means ± SEMs as percentages of the control values. Six independent ADP phosphorylation assays were performed, and fluorescence was measured on 20 cells. ∗p = 0.001, ∗∗p = 0.0001, ∗∗∗p < 0.0001 upon Student’s t-tests. GFP was measured with the 488 nm laser line and 505–550 nm long-pass filter. [file mmc10.pdf]

Suppl Fig. 2

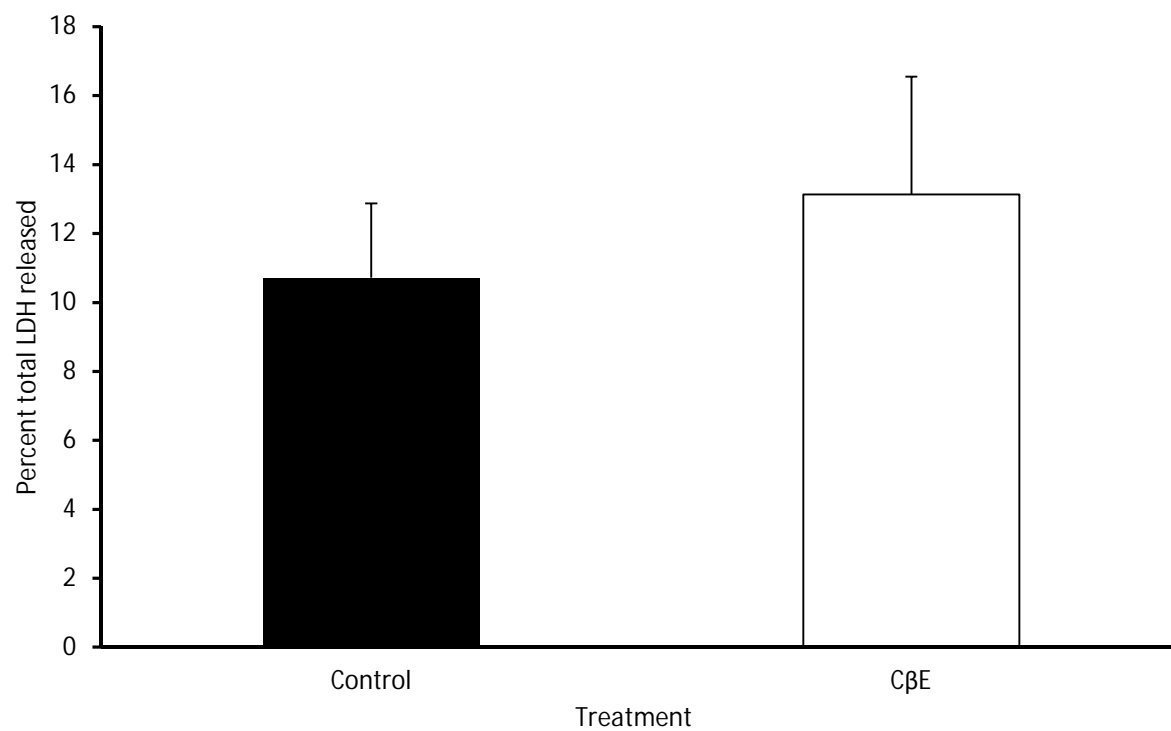

Supplement: Supplementary data 2 — CβE treatment had no significant effect on cell viability as judged by LDH release. Following CβE treatment for 30 days, cells were assessed for cell death by the LDH assay (solid bar: control, open bar: treated). No significant difference was seen between control and CβE cells. [file mmc2.pdf]

Suppl Fig.3

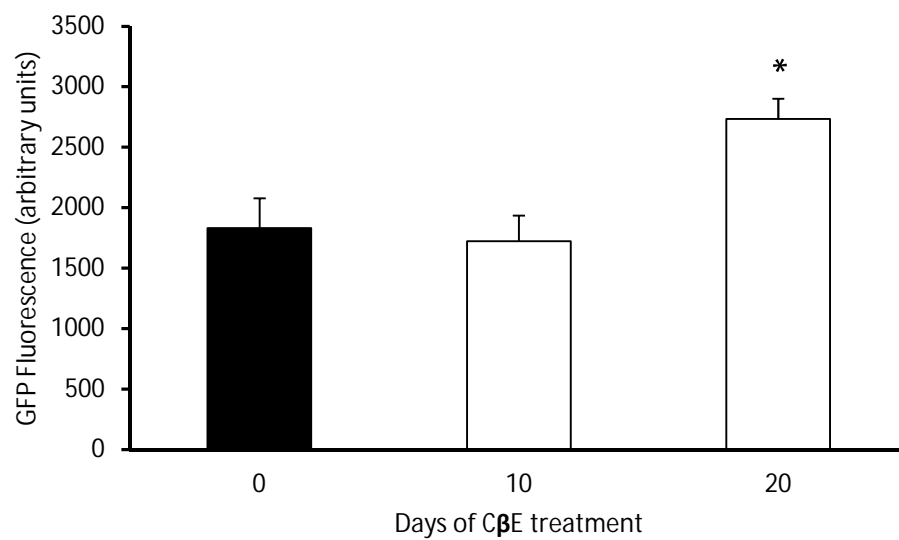

Supplement: Supplementary data 3 — CβE treatment led to accumulation of alpha-synuclein. Stable ectopic expression of a GFP-alpha-synuclein fusion (kindly provided by Dr. J. George, University of Illinois) was established in SHSY-5Y cells, and these cells were treated with 10 μM retinoic acid for 5 days, followed by 50 μM CβE for 10 and 20 days. GFP fluorescence was measured from 7 cells, and the mean ± SEM are shown. Statistical significance (∗p = 0.01) was found in GFP fluorescence between the 20 days CβE treatment (open bar on the right) and no treatment (solid bar), by Student’s t-tests. [file mmc3.pdf]

Suppl Fig. 4A

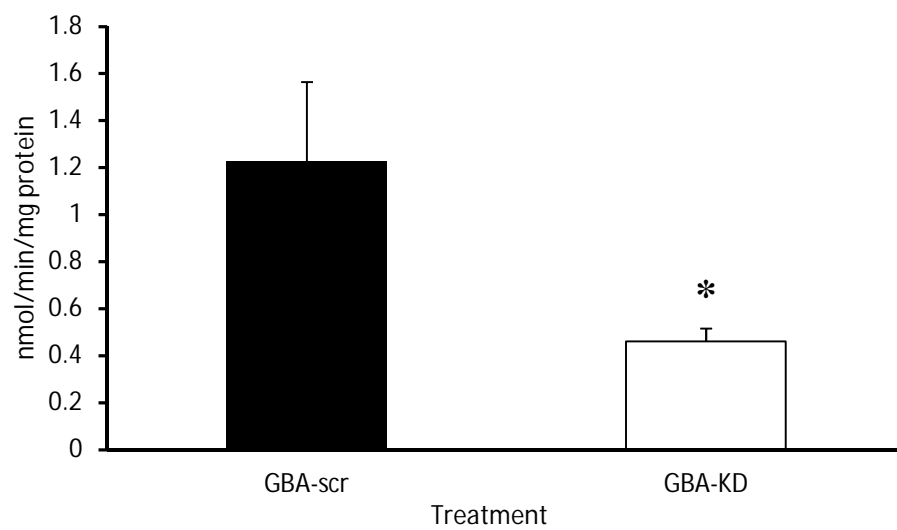

Supplement: Supplementary data 4 — GBA knockdown led to a fall in mitochondria membrane potential (ψm) and decreased aconitase activity. (A): GCase activity, as measured for GBA scrambled shRNA control (GBA-scr, solid bar) and a knockdown sequence (GBA–KD, open bar); a statistically significant reduction in GCase activity was seen with the knockdown (∗p < 0.02; n = 3). [file mmc4.pdf]

Suppl Fig. 4B

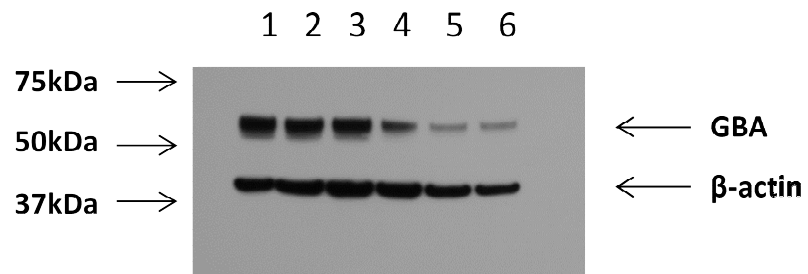

Suppl Fig. 4C

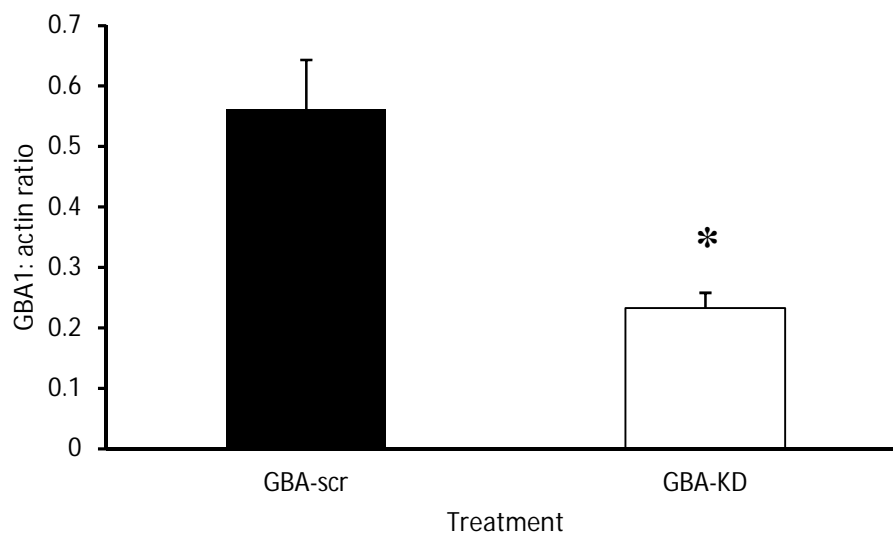

Supplement: Supplementary data 5 — GBA knockdown led to a fall in mitochondria membrane potential (ψm) and decreased aconitase activity. (B): Protein levels were estimated by Western blot, using GBA antibody and β-actin antibody as a loading control. Upper bands: GBA, lower bands: β-actin; bands 1–3 GBA-scr, bands 4–6 GBA–KD. (C): Shows a graphical representation of the blot (solid bar: scrambled control, open bar: knockdown), the GBA–KD being reduced significantly (∗p < 0.003, n = 3). [file mmc5.pdf]

Suppl Fig. 4D

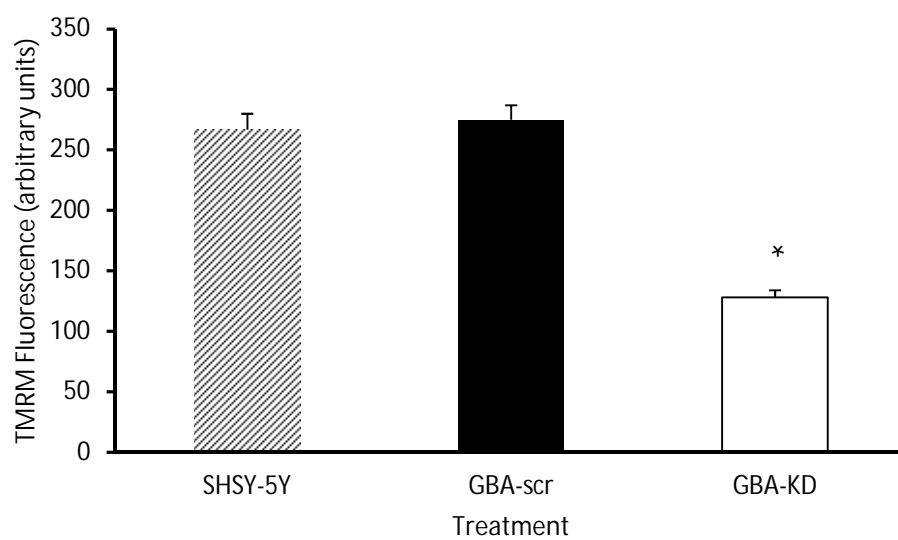

Supplement: Supplementary data 6 — GBA knockdown led to a fall in mitochondria membrane potential (ψm) and decreased aconitase activity. (D): Control SHSY-5Y (diagonal bars), GBA-scr (solid bars) and GBA–KD cells (open bars), each 60 cells from 3 coverslips representing 2 independent preparations, were analysed to produce the mean ± SEM TMRM fluorescence. Upon ANOVA followed by Tukey-Kramer Multiple Comparisons Test, that of GBA–KD is statistically significant (∗p < 0.05) compared to the other 2 controls. [file mmc6.pdf]

Suppl Fig. 4E

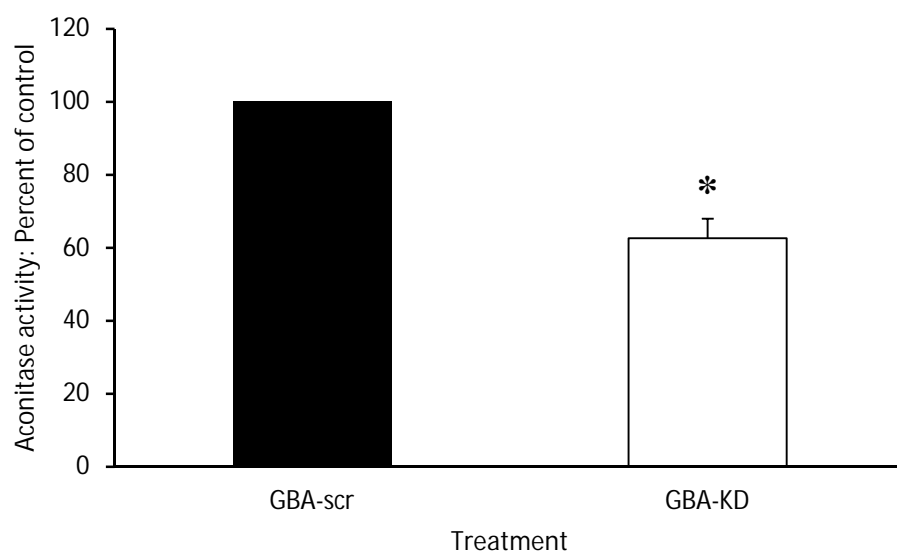

Supplement: Supplementary data 7 — GBA knockdown led to a fall in mitochondria membrane potential (ψm) and decreased aconitase activity. (E): Influence of reduction in GCase activity on aconitase activities in GBA-scr (solid bars) and GBA–KD cells (open bars). The activity of aconitase was reduced compared to control by a statistically significant amount of 38% (∗p = 0.003, n = 3). [file mmc7.pdf]

Suppl Fig. 5A

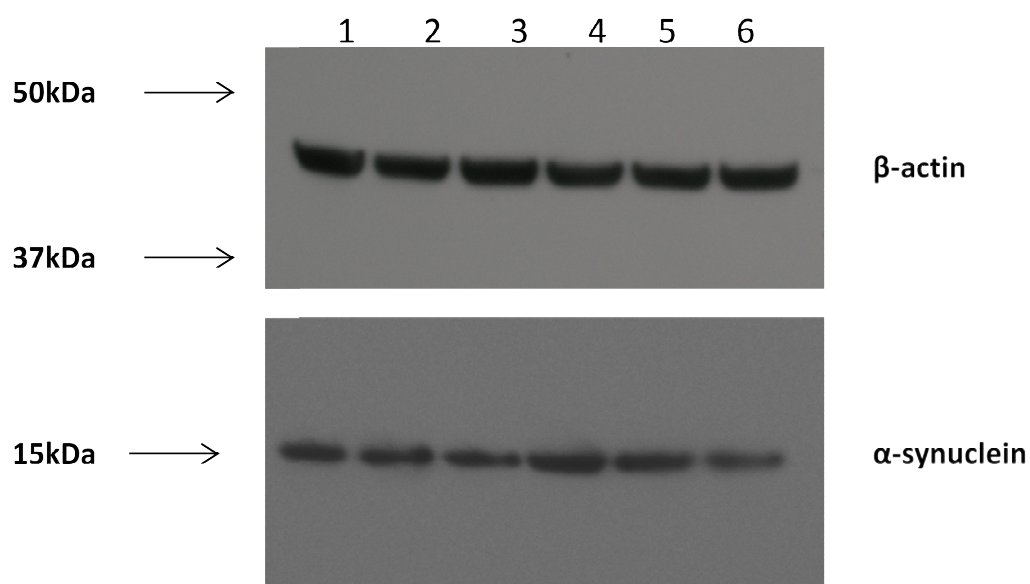

Suppl Fig. 5B

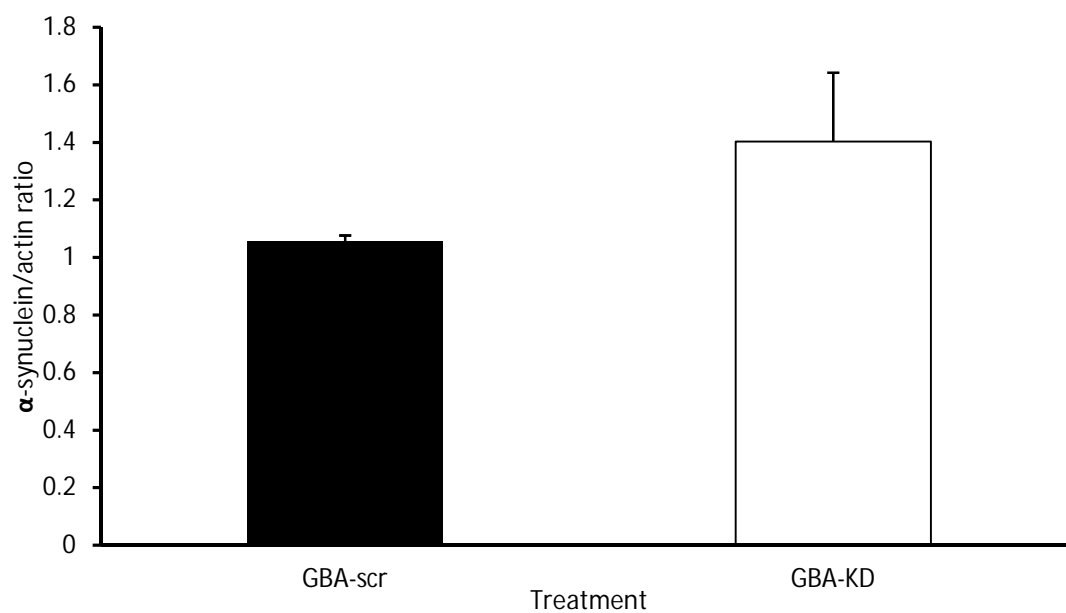

Supplement: Supplementary data 8 — GBA knockdown effect on alpha-synuclein levels. Levels of alpha-synuclein were measured in SHSY-5Y GBA knockdown and scrambled cells by Western blot analysis. (A) Shows the blots of scrambled control (GBA-scr, lanes 1–3) or knockdown (GBA–KD, lanes 4–6) cells, stained with anti-alpha synuclein (lower panels) or anti-β-actin (upper panels). (B) Shows the graphical representation of alpha-synuclein levels from the scanned blots, normalised to β-actin levels (solid bars: scrambled control, open bars: knockdown). The level of alpha-synuclein protein was non-significantly (p < 0.067; n = 3) higher (by 32%) over that of control. [file mmc8.pdf]

Suppl Fig.6A

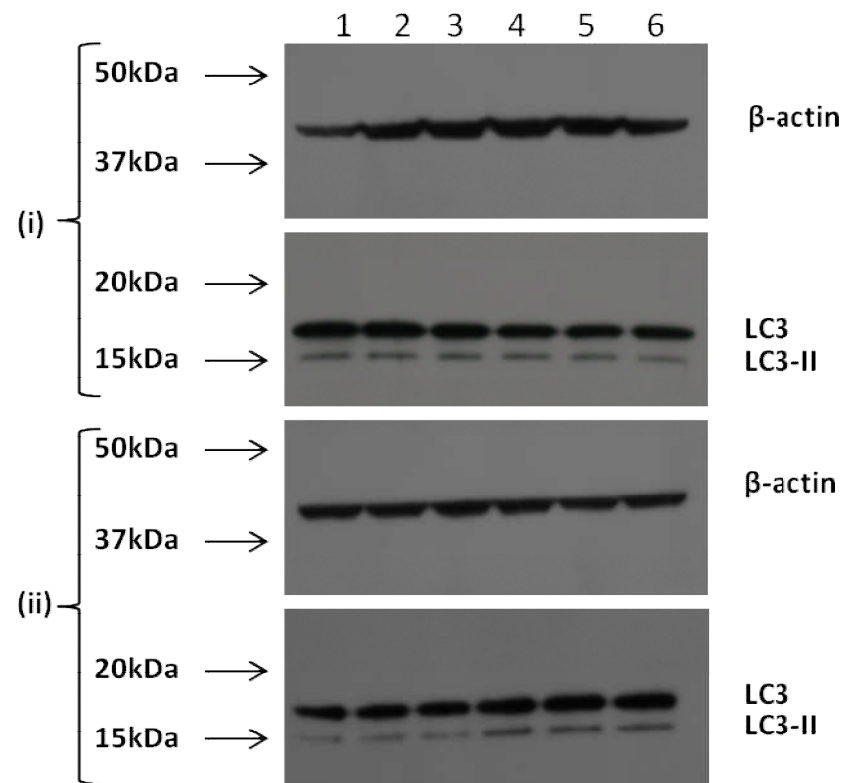

Suppl Fig.6B

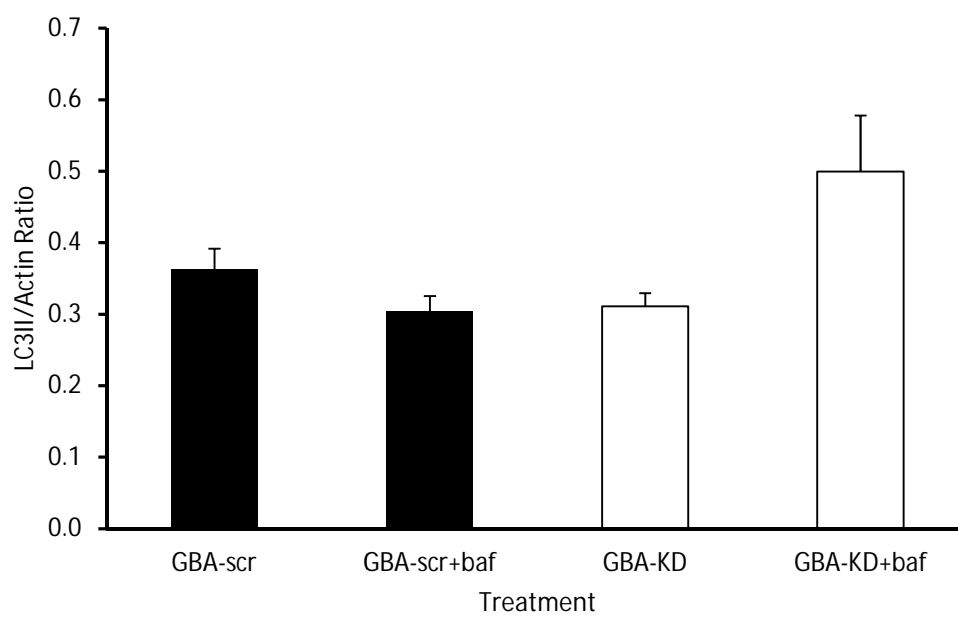

Supplement: Supplementary data 9 — Basal LC3Il levels for GBA–KD were not significantly changed by GBA knockdown. LC3-II levels were estimated for knockdown and control cells. (A): Knocking down GCase activity did not affect the basal levels of LC3-II; (blot (i): without bafilomycin; blot (ii): with bafilomycin); control (GBA-scr, lanes 1–3) or knockdown (GBA–KD, lanes 4–6). (B) Shows a graphical representation of the blots (solid bars: scrambled control, open bars: knockdown). [file mmc9.pdf]
